# Vegetarian diet and mental disorders: results from a representative community survey

**DOI:** 10.1186/1479-5868-9-67

**Published:** 2012-06-07

**Authors:** Johannes Michalak, Xiao Chi Zhang, Frank Jacobi

**Affiliations:** 1 Department of Clinical Psychology, University of Hildesheim, 31141 Hildesheim, Germany; 2 Department of Clinical Psychology, Ruhr-Universität Bochum, Germany; 3 Psychologische Hochschule Berlin and Institute of Clinical Psychology and Psychotherapy, Germany; 4 Center of Epidemiology and Longitudinal Studies (CELOS), Technische Universität, Dresden, Germany

**Keywords:** Vegetarian diet, Psychopathology, Epidemiology

## Abstract

**Background:**

The present study investigated associations between vegetarian diet and mental disorders.

**Methods:**

Participants were drawn from the representative sample of the German Health Interview and Examination Survey and its Mental Health Supplement (GHS-MHS). Completely vegetarian (N = 54) and predominantly vegetarian (N = 190) participants were compared with non-vegetarian participants (N = 3872) and with a non-vegetarian socio-demographically matched subsample (N = 242).

**Results:**

Vegetarians displayed elevated prevalence rates for depressive disorders, anxiety disorders and somatoform disorders. Due to the matching procedure, the findings cannot be explained by socio-demographic characteristics of vegetarians (e.g. higher rates of females, predominant residency in urban areas, high proportion of singles). The analysis of the respective ages at adoption of a vegetarian diet and onset of a mental disorder showed that the adoption of the vegetarian diet tends to follow the onset of mental disorders.

**Conclusions:**

In Western cultures vegetarian diet is associated with an elevated risk of mental disorders. However, there was no evidence for a causal role of vegetarian diet in the etiology of mental disorders.

## Background

A small but increasing number of people in Western countries are choosing to restrict meat for various reasons. While in countries such as India a high proportion (35%) of the population follows a vegetarian diet due to cultural and religious traditions [1], rates in Western countries are much lower. However, a considerable minority of populations in Western countries do not consume meat. In a methodologically sound study [2] 1.6% of respondents in a representative sample of 20.000 Germans reported being vegetarians [2]. Estimates for US and UK samples are slightly higher (3%) [3,4].

During the past decades, increasing knowledge has emerged about the effects of vegetarian diet on nutritional status and physical health. Taken as a whole, studies have shown that vegetarians are in good physical health compared with national averages and as healthy as non-vegetarians with a comparable background and lifestyle [5,6]. This outcome can be explained by the more health-conscious behaviors of vegetarians and by the fact that vegetarian diets are often healthy with the respect to such factors as fat composition [7] and fiber [8].

Although our knowledge about the association between vegetarian diet and physical health is based on numerous studies, relatively little data is available on the associations between vegetarian diet and mental health. Diverse processes could in principle produce differences between vegetarians and non-vegetarians in rates of mental disorders. On a biological level, nutrition status resulting from vegetarian diet may affect neuronal function and synaptic plasticity, which in turn influences brain processes relevant for onset and maintenance of mental disorders [9,10]. For example, there is strong evidence that long-chain n-3 fatty acids causally affect risk for major depressive disorders [11,12]. Moreover, although evidence is less unequivocal, vitamin B_12_ levels appear to be causally linked to major depressive disorders. Studies have reported that vegetarians show lower tissue concentrations of long-chain n-3 fatty acids [13,14] and vitamin B_12_[15,16] which may elevate risk for major depressive disorder.

Besides differences in nutrition status, vegetarians and non-vegetarians differ in a number of psychological and socio-demographic characteristics that may influence their risk for mental disorders. Vegetarians are predominantly female [17], are more likely to live in urban areas and to be single [18]. All these socio-demographic factors are correlates of the presence of mental disorders [19]. Moreover, vegetarians tend to be more aware of the factors influencing their dietary intake and of the importance of a healthy lifestyle in general. A number of studies have shown that vegetarians tend to be slimmer, drink less alcohol, and exercise more than non-vegetarians [20-23]. In addition, vegetarians tend to define themselves negatively by emphasizing what they do not do; they tend to stress their dissimilarity from others and thereby accentuate their differences from the general society [24]. Moreover, some vegetarians base the choice of their diet more on an ethical motivation [25]. Thus, some psychological and socio-demographic characteristics of vegetarians, such as negative self-definition and dysfunctional eating attitudes, could have detrimental effects on mental health; other characteristics could have beneficial effects, such as a healthy lifestyle and ethical motivation.

In summery, any associations that may be found between vegetarian diet and mental disorders could be attributable to several possible causal mechanisms: (a) the biological effects of diet have an influence on brain processes that increases the chance for the onset of mental disorders, in which case it could be expected that adopting a vegetarian diet would precede the onset of mental disorders; (b) relatively stable psychological characteristics independently influence the probability of choosing a vegetarian diet pattern, and developing a mental disorder, in which case the adoption of the diet and the onset of a mental disorder would be unrelated; or (c) developing a mental disorder increases the likelihood of choosing a vegetarian diet, in which case the onset of the mental disorder would precede the vegetarian diet. Although published findings on that type of relationship are missing, it is conceivable that individuals with mental disorders are more aware of suffering of animals or may show more health-oriented behaviors (e.g. adopting a vegetarian diet) in order to positively influence the course of their mental disorder.

Few empirical studies have directly tested associations between vegetarian diet patterns and mental health. We could locate only seven studies of mental health in vegetarians which all were limited in their measures of mental health, as they were based only on self-report, and with no formal diagnosis. Moreover, participants were not broadly representativeness of the community population, because they were either adolescents, young adults, or drawn from a special population. Five of seven studies analyzed characteristics of vegetarian adolescents compared to non-vegetarian adolescents: (a) Using a single item measure of depression, Larsson et al. [17] found that vegetarian adolescents were more often depressed during the previous week; (b) in the study of Perry, McGuire, Neumark-Sztainer, and Story [26] vegetarian adolescents were more likely to have contemplated and attempted suicide (assessed with a single item) than non-vegetarians. However, no significant differences were found in current depression symptoms (assessed with a 7-item scale) between the groups. Furthermore, vegetarians more frequently reported having been told by a physician that they had an eating disorder; (c) in another study by Neumark-Sztainer, Story, Resnick, and Blum, adolescent vegetarians reported more deviant eating behaviors, including higher rates of dieting, intentional vomiting and laxative use (all single item measures) [27]; (d) in addition, Bas, Karabudak and Kiziltan [28] found abnormal eating attitudes, low self-esteem, high social anxiety, and high trait anxiety (all assessed with psychometrically sound self-report measures) in Turkish vegetarian adolescents; (e) in a study with adolescents and young adults (age: 15–23 years) Robinson-O'Brien, Perry, Wall, Story, and Neumark-Sztainer, [29] showed that current vegetarians may be at increased risk for binge eating with loss of control (assessed with two questions), while former vegetarians may be at increased risk for extreme unhealthful weight-control behaviors (assessed by asking participants whether they showed one of the following behaviors during the past year: “took diet pills,” “made myself vomit,” “used laxatives,” and “used diuretics”).

Baines et al. [18] investigated mental health in a representative sample of young (22–27 years old) Australian women. Vegetarians reported higher levels of depression during the previous 12 months (single item measure) and were more often diagnosed with depression by a doctor than were their non-vegetarian counterparts. It should be noted that vegetarian and non-vegetarian women also differed in socio-demographic characteristics (i.e., vegetarians were more likely to live in urban areas and to be single).

The only study we found to report better mental health in vegetarians was conducted by Beezhold, Johnston, and Daigle in a sample of Seventh Day Adventist (i.e., members of a Christian denomination) adults [30]. In this study, vegetarian Adventists reported significantly less negative emotions than did non-vegetarian Adventists, as assessed by the Depression Anxiety Stress Scales and the Profile of Mood States [31]. The discrepancy between this study and the previously cited studies may be due to the special population in the Beezhold et al. study of member of a small religious community, unlike participants in the other studies who were drawn from more general populations. Although not all Seventh Day Adventists are vegetarians, vegetarianism is highly valued and commonly practiced among Seventh Day Adventists, giving followers of this diet strong coherence and high status in their peer group. In contrast, most vegetarians in Western societies consciously have to reject the opinions of the majority (i.e., eating meat), an act possibly more isolating from than joining with others [24].

Previous studies on mental health of vegetarians have been restricted to specific groups, young age ranges, and mostly in people unrepresentative of the community. This poses a problem with regard to the average adult population, for whom dietary issues or vegetarian orientation may have a different significance compared to individuals of younger age. No study to date has investigated mental health in a representative adult sample encompassing a broad range of age groups. Furthermore, the studies so far are limited in the assessment of mental health exclusively by self-report questionnaires. No study to date has used clinical diagnoses of mental disorders based on standardized diagnostic interviews. In addition, most studies are limited to a set of specific behaviors (e.g., depressive symptoms, deviant eating behavior) and thus fail to provide a broader perspective on the mental health status of vegetarians in Western cultures.

The present study aimed to investigate associations between vegetarian diet and mental disorders in a representative sample of adults between 18 and 65 years of age. We used data from the German National Health Interview and Examination Survey and its Mental Health Supplement (GHS-MHS), a nationwide epidemiological study of both somatic and mental health in Germany in a representative community sample [32]. Extending previous research, we were interested in a broad spectrum of mental disorders (unipolar depressive disorders, anxiety disorders, somatoform disorders and syndromes, and eating disorders) which were assessed by a standardized individual face-to-face diagnostic interview for mental disorders by clinically trained interviewers according to the criteria in the Diagnostic and Statistical Manual of Mental Disorders (4th ed.; DSM-IV) [33]. Although the GHS-MHS was cross-sectional, participants’ retrospective age of onset of their mental disorders and of their vegetarian diet enabled us to investigate whether diet change precedes or follows the onset of mental disorders. In addition, we analyzed the consumption of various food products (meats, vegetables and fruits, fast food, fish) in individuals with different mental disorders. We expected that, consistent with previous studies on vegetarian diet and mental health, vegetarians would exhibit more mental disorders. We further predicted that people with mental disorders would have lower frequencies of meat intake.

## Methods

### Design and participants

Participants were 4,181 respondents to the German National Health Interview and Examination Survey (GHS) conducted in 1998/1999. The GHS was designed to provide representative nationwide epidemiologic data on major somatic and mental disorders, impairments, and healthcare utilization. The survey was approved by the institutional review board of the Robert Koch Institute (Berlin, Germany). All participants provided written informed consent (for details on the GHS see citation 32).

The GHS was based on a two-stage modular design and consisted of 1) the core survey (N = 7,124) with a wide range of physical assessments and comprehensive questionnaires about health related behaviors including a nutrition survey, and 2) the mental health supplement (GHS-MHS, N = 4,181) given to a subsample of those who completed the original core survey. The core survey sample was drawn from the population registries of people aged 18 to 79 living in Germany in 1997 (stratified random sample from 113 communities throughout Germany with 130 sampling units, response rate: 61.4%). Because the psychometric properties of the diagnostic interview (Composite International Diagnostic Interview, M-CIDI) [34-36] had not been established for use in older populations at that time [37], the subsample of the GHS-MHS (conditional response rate: 87.6% from N = 4,775 eligible participants) was restricted to respondents up to 65 years of age. This subsample was representative of the German non-institutionalized adult population aged between 18 and 65 years of age.

### Assessment of mental disorders

Psychiatric diagnoses were assessed by the computer-assisted version of the Munich Composite International Diagnostic Interview (M-CIDI). This fully structured interview is a modified version of the World Health Organization CIDI, version 1.2 [38] supplemented by questions to cover DSM-IV criteria. Because the use of lay interviewers has been discussed as a limitation of CIDI studies in the past, interviews in the present study were conducted by clinically trained interviewers (psychologists and physicians) with an average interview duration of 65 minutes. Psychometric properties of the M-CIDI were found to range from acceptable to very good for all diagnoses of the current study: the test–retest reliability ranged from kappa = 0.45 for Generalized Anxiety Disorder to 1.00 for Panic Disorder.

One-month, 12-month and lifetime prevalence rates as well as age of onset data for the following diagnostic groups were assessed:

a) *Depressive disorders*: Major depressive disorder, dysthymic disorder;

b) *Anxiety disorders*: Panic disorder (with or without agoraphobia), agoraphobia without a history of panic disorder; specific phobias, social phobia; obsessive-compulsive disorder, generalized anxiety disorder;

c) *Somatoform disorders and syndromes*: Somatization disorder, “abridged somatization disorder” (a less stringent defined somatization syndrome than DSM Somatization Disorder) [39], hypochondriasis and pain disorder.

d) *Eating disorders*: Anorexia nervosa, bulimia nervosa (including atypical anorexia nervosa and bulimia nervosa)

### Diet pattern

Within the nutrition-survey that was part of the core survey a vegetarian diet pattern was assessed with the item ‘*Do you currently follow a vegetarian diet (no meat) or did you follow a vegetarian diet in the past?’* Participants could answer either ‘*no, never’*, ’*yes, completely’*, or ’*yes, predominantly’.* It should be noted that the word ‘meat’ in German language excludes poultry. Additionally, vegetarian participants were asked to indicate the age at which they adopted a vegetarian diet.

Furthermore the consumption pattern of food products within the past 12 months was assessed with the question ‘*How often do you consume the following food products (from a list of 35 items)? Please consider the past 12 months*.’ Participants rated each food on a 7-point scale: 0 = *almost never* 1 = *once a month or less frequently;* 2 = *twice or three times a month*; 3 = *approximately once a week;* 4 = *several times a week;* 5 = *daily or almost daily;* 6 = *several times a day*. We analyzed associations between the presence of mental disorders and consumption of a) meats (*meat and processed meats);* b) vegetables and fruits (*green salads, raw vegetable salads, raw vegetables, fresh fruits*); c) fast food (*bratwurst, hamburger, pizza, döner kebab*); d) *fish.*

In the sample, 1.3% (n =  54) of the participants reported following exclusively and 4.5% (n = 190) predominantly vegetarian diets. We also examined whether the consumption of fish has an effect on the pattern of results. We computed the percent of people in the exclusively and predominantly vegetarian group who did not consume fish. No fish was consumed during the past 12 months by 48.2% of the exclusively and 14.7% of the predominantly vegetarian group. Because results for vegetarians with and without fish consumption were very similar, we collapsed results of both groups. 92.3% of the sample (n = 3872) was non-vegetarian or information about their vegetarian status was missing (1.6%, n = 65; these were excluded from the analyses).

### Statistical analysis

We first calculated rates of mental disorders of completely vegetarians, predominantly vegetarians and non-vegetarian participants (total sample). Vegetarians tend to differ from non-vegetarians in socio-demographic characteristics associated with the prevalence of mental disorders. We therefore included an additional stratified sampling matching procedure to obtain a comparison group of non-vegetarian participants with socio-demographic characteristics matched to the completely and predominantly vegetarian subsample. The total sample of non-vegetarians was divided into strata by five socio-demographic characteristics: sex, age, educational level, the size of the community (< 20.000, 20.000 to 100.000, or > 100.000) and marital status (‘single’, ‘married’, ‘separated, divorced, widowed’). Then a random sample was taken from each stratum and the samples were pooled to yield a non-vegetarian ‘control group’ with socio-demographic characteristics similar to those in the vegetarian group. The matched control group (N = 242) consisted of all the proportional samples.

In testing our main hypothesis, we considered only the differences in disorder prevalence rates between vegetarians and the matched control group of non-vegetarians, for which we used odds ratios (OR, 95% CI).

To compare consumption of food products for participants with and without a mental disorder, we conducted t-tests for each of the four diagnostic groups (depressive disorders, anxiety disorders, somatoform disorders and syndromes, eating disorders).

To determine whether adoption of the vegetarian diet precedes or follows onset of mental disorders we computed a difference score between the age at adoption of the vegetarian diet and the age at onset of mental disorder. Then we tested, for each mental disorder, whether the difference scores significantly deviated from 0 (independent one-sample t-test). A negative score indicates that the vegetarian diet came first, a positive score that the mental disorder came first.

## Results

### Demographic comparison of vegetarians and non-vegetarians

Socio-demographic characteristics of the completely vegetarian, predominantly vegetarian, total non-vegetarian, and matched non-vegetarian samples are shown in Table 1.

**Table 1:** Socio-demographic characteristics of the completely vegetarian, predominantly vegetarian, non vegetarian total and non vegetarian matched sample

	**Completely vegetarian**	**Predominantly vegetarian**	**Non vegetarian (total sample)**	**Non vegetarian (matched sample)**
*N/%* female	54/ 70.4%	190 / 75.3%	3872 / 53.0%	242 / 74.0%
Age (*SD*)	35.4 (13.6)	39.3 (13.4)	41.9 (13.0)	39.0 (13.3)
Secondary graduation (Abitur), % (N)	37.0% (20)	36.8% (70)	15.9% (617)	32.2% (78)
Community size % (N)				
pop. < 20.000	29.6% (16)	31.6% (60)	45.6% (3016)	31.4% (76)
pop. = 20.000-100.000	31.5% (17)	24.7% (47)	25.2% (1664)	25.6% (62)
pop. > 100.000	38.9% (21)	42.7% (83)	29.3% (1936)	43.0% (104)
Living situation: Single; % (N)	51.9% (28)	36.3% (69)	23.1% (893)	40.1% (97)
Married, % (N)	33.0% (18)	46.8% (89)	64.7% (2505)	44.2% (107)
Separated, divorced, widowed, % (N)	14.9% (8)	16.4% (31)	11.6% (453)	15.7% (38)

pop. = population.

The vegetarian sample (completely plus predominantly vegetarians) differed from the non-vegetarian total sample in several socio-demographic variables. Vegetarians were younger (*t* = 4.01; *df* = 4114, *p* < .001), predominantly female (χ^2^.[1, *N* = 4116] = 41.41, *p* < .001), more likely not to be married (χ^2^.[2, *N* = 4094] = 45.70, *p* < .001), to live in urban areas (χ^2^.[2, *N* = 4116] = 25.34, *p* < .001) and to have advanced high school education (Abitur) (χ^2^.[3, *N* = 4098] = 62.82, *p* < .001). The matching procedure was successful, i.e. this subsample statistically no longer differed from the vegetarian sample in any of the socio-demographic variables (all *p*s > .95).

### Rates of mental disorders in vegetarians

Shown in Table 2 are the 1-month, 12-month and lifetime prevalence rates of mental disorders in the completely vegetarian, predominantly vegetarian, non-vegetarian total, and in non-vegetarian matched samples. The 12-month and lifetime prevalence rates of depressive disorders were elevated in the completely vegetarian group. Prevalence rates of the complete vegetarians are nearly 15% higher than of the non-vegetarians. Prevalence rates of individuals with a predominantly vegetarian diet were between the rates of the completely vegetarian and the non-vegetarian group.

**Table 2:** Prevalence rates of mental disorders in the completely vegetarian, predominantly vegetarian, non vegetarian (total and matched) sample

	**Completely Vegetarian (N=54)**	**Predominantly Vegetarian (N=190)**	**Non vegetarian (total sample) (N=3872)**	**Non vegetarian (machted sample) (N=242)**	**Comparision completely/ predominatly vs. matched non-vegetarians OR (95% CI)**	**Comparision completely vs. matched non-vegetarians OR (95% CI)**
Unipolar Depressive Disorders						
1-month	7.4%	6.8%	6.3%	5.0%	1.44 (0.67-3.07)	1.53 (0.48-4.95)
12-month	24.1%	14.7%	11.9%	10.3%	1.75 (1.03-2.99)	2.75 (1.30-5.82)
lifetime	35.2%	25.8%	19.1%	20.7%	1.48 (0.98-2.26)	2.09 (1.10-3.95)
Anxiety Disorders						
1-month	20.4%	12.6%	10.7%	8.7%	1.76 (0.99-3.13)	2.69 (1.12-5.99)
12-month	31.5%	19.5%	17.0%	13.2%	1.87 (1.15-3.01)	3.02 (1.52-5.98)
lifetime	31.5%	22.1%	18.4%	15.3%	1.77 (1.12-2.79)	2.55 (1.30-4.99)
Somatoform Disorders and Syndromes						
1 month	9.3%	12.1%	7.8%	4.5%	2.72 (1.32-5.60)	2.14 (0.71-6.44)
12 month	16.7%	17.9%	11.6%	9.5%	2.04 (1.19-3.50)	1.90 (0.83-4-39)
lifetime	25.9%	25.8%	16.9%	15.3%	1.93 (1.23-3.03)	1.94 (0.96-3.91)
Eating Disorders						
1-month	3.7%	1.1%	0.1%	0.0%	(not computable)	
12-month	3.7%	1.6%	0.3%	0.8%		
lifetime	5.6%	3.2%	0.6%	1.2%		

CI = confidence interval; OR = odds ratio.

Moreover, vegetarians consistently showed higher rates of anxiety disorders. This pattern was evident for 1-month, 12-month, and lifetime prevalence rates. Only the comparison of 1-month prevalence rates between the completely/predominantly vegetarian groups versus the non-vegetarian group showed a marginal trend. All other comparisons were statistically significant (at *p* < .05). Prevalence rates for anxiety disorder were especially high (more than twice as high) in the completely vegetarian group than in the non-vegetarian matched group.

For somatoform disorders and syndromes, 1-month, 12-month and lifetime prevalence rates were statistically significantly elevated in the completely/predominantly vegetarians compared to the non-vegetarians. However, when only the complete vegetarians were compared to the non-vegetarians, we did not find statistically significant differences in prevalence rates of somatoform problems. Prevalence rates of somatoform disorders and syndromes in completely and predominantly vegetarians were quite similar, and were 5%-10% higher than in the non-vegetarians.

For eating disorders we can report only prevalence rates but not ORs or CIs for the vegetarian and non-vegetarian groups, because eating disorders were too rare (n = 36 in total sample, and n = 6 in the vegetarian group). A minimum of 10 events per independent variable is recommended in a logistic regression [40]. Otherwise there is a tendency to systematically overestimate ORs. However, descriptively we found strongly elevated 1-month, 12-month and lifetime prevalence rates in the completely vegetarian group.

### Kinds of food eaten by participants with and without mental disorders

Consumption of food products by individuals with and without mental disorders is shown in Table 3. A very consistent pattern emerged for meat consumption: individuals suffering from a depressive, anxiety, or eating disorder as well as from a somatoform disorder and syndrome consumed less meat. This pattern emerged for 1-month, 12-month as well as lifetime prevalence rates. Analysis in the subsample of non-vegetarians also consistently showed that people suffering from a mental disorder ate less meat than people without a mental disorder. For vegetables and fruits, fast food, and fish a less consistent pattern emerged, although there are view findings showing those without mental disorders to eat more of all of these kinds of food, as shown in Table 3.

**Table 3:** Mean consumption of food products as self-rated on a scale ranging from 0 (“almost never”) to 6 (consume it “several times a day”) in individuals with (+) and without (−) selected mental disorders

**Disorder**	***with (+) without (−) disorder***	**Meats**			**Vegetables and fruits**			**Fast food**			**Fish**		
		***M***	***SD***	***t (df)***	***M***	***SD***	***t (df)***	***M***	***SD***	***t (df)***	***M***	***SD***	***t (df)***
Depressive Disorders													
1 month	+	3.72	1.07	2.87**	4.19	1.11	0.93	1.58	1.14	1.99*	2.27	1.24	0.36
	-	3.92	1.05	(4111)	4.26	0.96	(288.62)	1.72	1.11	(4109)	2.30	1.17	(293.49)
12 month	+	3.70	1.14	4.39***	4.21	1.04	1.07	1.56	1.14	3.30**	2.20	1.23	2.00*
	-	3.93	1.04	(624.50)	4.26	0.96	(627.36)	1.73	1.11	(4109)	2.32	1.16	(631.31)
lifetime	+	3.68	1.17	6.29***	4.29	0.97	1.14	1.56	1.10	4.38***	2.23	1.22	1.87
	-	3.96	1.02	(1128.80)	4.25	0.97	(1215.38)	1.75	1.11	(4109)	2.32	1.16	(1189.21)
Anxiety Disorders													
1 month	+	3.74	1.12	3.38**	4.14	1.10	2.39*	1.73	1.14	0.50	2.14	1.30	2.90**
	-	3.92	1.04	(548.62)	4.27	0.96	(536.25)	1.71	1.11	(4109)	2.32	1.15	(539.36)
12 month	+	3.73	1.12	4.66***	4.17	1.07	2.32*	1.67	1.13	1.12	2.18	1.13	2.85**
	-	3.94	1.04	(983.14)	4.27	0.95	(965.86)	1.72	1.11	(4109)	2.32	1.14	(959.09)
lifetime	+	3.75	1.04	4.44 ***	4.19	1.06	2.02*	1.64	1.13	2.03*	2.21	1.28	2.35*
	-	3.94	1.11	(1105.96)	4.27	0.95	(1082.94)	1.73	1.11	(4109)	2.32	1.14	(1075.26)
Somatoform Disorders and Syndromes													
1 month	+	3.66	1.18	4.01***	4.27	0.98	0.31	1.60	1.08	1.87	2.30	1.26	0.18
	-	3.93	1.04	(377.29)	4.25	0.97	(4109)	1.72	1.11	(4109)	2.30	1.16	(382.20)
12 month	+	3.72	1.19	3.76***	4.30	0.98	1.13	1.67	1.14	0.76	2.30	1.16	0.01
	-	3.93	1.03	597.34	4.25	0.97	(4109)	1.71	1.11	(4109)	2.30	1.24	(616.20)
lifetime	+	3.72	1.19	4.63***	4.27	1.02	0.36	1.59	1.10	3.17**	2.32	1.18	0.47
	-	3.95	1.02	(955.51)	4.26	0.97	(4109)	1.73	1.11	(4109)	2.30	1.16	(4106)
Eating Disorders													
1 month	+	1.39	1.39	7.21***	4.83	0.55	1.79	0.89	0.74	2.22*	1.67	1.23	1.63
	-	3.91	1.05	(4111)	4.25	0.97	(4109)	1.71	1.11	(4109)	2.30	1.17	(8.03)
12 month	+	2.29	1.82	3.57**	4.47	0.88	0.88	1.28	1.05	1.54	2.19	1.42	0.39
	-	3.91	1.04	(15.04)	4.26	0.97	(4109)	1.71	1.11	(4109)	2.30	1.17	(4106)
lifetime	+	3.18	1.77	2.41*	4.19	1.06	3.77	1.59	1.26	0.63	2.03	1.19	1.36
	-	3.19	1.04	(33.10)	4.25	0.97	(4109)	1.71	1.11	(4109)	2.30	1.17	(4106)

* p < .05, ** p < .01, *** p < .001 two-tailed.

### Adoption of a vegetarian diet and age of onset of mental disorders

The mean ages of onset of mental disorders in vegetarians and adoption of a vegetarian diet are shown in Table 4. Results of t-tests for the difference score are depicted in Table 5. For depressive and anxiety disorders, as well as somatoform disorders and syndromes, the results of the t-tests indicate that on average the start of a vegetarian diet follows the onset of mental disorder. The normal distribution of the difference scores and visual inspection of the data indicate that this result is not attributable to outliers. For eating disorders we found no significant deviation of the mean difference score from 0. We also computed the percentage of vegetarians starting a vegetarian diet before the onset of mental disorders. This proportion was 34.4% for depressive disorders; 9.3% for anxiety disorders, 18.3% for somatoform disorders and syndromes and 50% for eating disorders.

**Table 4:** Mean age at adoption of the vegetarian diet and at the onset of mental disorder in completely and predominantly vegetarians with mental disorder

**Age of onset**	***N***	***M***	***SD***
Vegetarian diet	121^a^	30.58	14.63
Depressive Disorders	68	24.69	12.95
Anxiety Disorders	62	18.85	13.01
Somatoform Disorders and Syndromes	63	19.04	10.36
Eating Disorders	6	16.00	2.00

^a^: number of vegetarians with mental disorders.

**Table 5:** Descriptive statistics for difference scores of age of onset data and results of t-tests in completely and predominantly vegetarians with mental disorder

**Disorders**	***M*^*a*^ Δ adoption vegetarian diet and onset of disorder, in years**	***SD***	***t (df)***	**Kolmogorov–Smirnov test**
Depressive Disorders	5.56	13.78	3.23 (63)**	1.09*ns*
Anxiety Disorders	10.26	12.26	6.15 (53)***	1.31*ns*
Somatoform Disorders and Syndromes	10.83	16.90	4.97 (59)***	0.84ns
Eating Disorders	−1.00	7.37	−0.33 (5) *ns*	0.43*ns*

^a^ positive values indicate that onset of mental disorder preceded adoption of the vegetarian diet.

* p < .05, ** p < .01, *** p < .001 two-tailed.

## Discussion

The major aim of our study was to investigate associations between a vegetarian diet and mental disorders in an epidemiological, community-based sample. The proportion of participants in the total sample who reported that they adhere to a completely vegetarian diet (i.e. no meat consumption) was 1.3%. A predominately vegetarian diet (predominantly consuming no meat) was followed by 4.6% of the total sample. The remaining 94.1% never adopted a vegetarian diet. These numbers are well in line with results of the large-scale study on diet by the Max Rubner Institut [2]. We found evidence for elevated prevalence rates in vegetarians for depressive disorders, anxiety disorders, somatoform disorders and syndromes as well as for eating disorders. It is important that such higher rates cannot be explained by different socio-demographic characteristics (e.g., 70% vegetarians were females, and females show higher base rates than men for these disorders). For this reason we designed a non-vegetarian control group that was matched with variables known to be associated with mental disorders (sex, age, educational level, marital status, urban residency). When compared to the non-vegetarian matched comparison sample, the vegetarian group showed even greater differences in the prevalence of mental disorders than when compared to the entire non-vegetarian sample.

The elevated prevalence rates of disorders in vegetarians are consistent with most previous studies. However, while samples of previous studies were restricted to specific groups and to mostly young cohorts, our study is the first to investigate this association in a representative adult sample. Moreover, our study is the first one to use standardized and comprehensive assessment of DSM-IV mental disorders and to analyze the temporal sequencing of the start of vegetarian diet and the age of onset of mental disorders.

Remarkably, in contrast to 12-month and lifetime prevalence rates, 1-month prevalence rates for depressive disorders were not elevated in vegetarians. This pattern in depressive disorders contrasts with the pattern observed in other types of disorders investigated in our study, where elevated 1-month prevalence rates were evident. However, it shows that the results of our study are not likely to be distorted by a memory bias caused by current depressive mood.

While the assessment of the vegetarian diet pattern in our study was independent of the time frame of the diet (i.e., the vegetarian item assessed a current as well as a past vegetarian diet), the data on the consumption of food products had a specific time reference (consumption within the past 12 months). Our analysis revealed that individuals suffering from mental disorders consistently showed lower frequencies of meat consumption during the past 12 months. These results again indicate that a current vegetarian or low meat consumption diet pattern is associated with elevated prevalence rates of mental disorders.

Consumption of other food products is less closely associated with mental disorders. For vegetables and fruits, foods associated with good physical health [41], we found reduced consumption only in individuals suffering from an anxiety disorder. In depressive disorders, somatoform disorders and syndromes as well as in eating disorders, we found no differences in the consumption of vegetables and fruits between individuals with or without a mental disorder. In contrast to results from a recent study showing a positive association between depressive symptoms and self-reported fast-food intake in midlife women [42] we found reduced rates of fast food consumption in individuals suffering from depressive disorders (1-month, 12-month as well as life-time prevalence rates). Moreover, people with a lifetime diagnosis of an anxiety disorder or somatoform disorder and syndrome also showed lower consumption of fast food. This pattern may indicate that individuals with mental troubles try to avoid food with potentially adverse health consequences to benefit their condition. Alternatively, a third variable (e.g., neuroticism, perfectionism) may cause both low consumption of fast food and increased vulnerability to mental disorders.

Fish consumption was most clearly (negatively) associated with anxiety disorders. This finding is in line with recent studies on associations between long-chain n—3 fatty acids and anxiety disorders [43,44]. Also individuals suffering from a depressive disorder during the past 12 months showed lower levels of fish consumption. This pattern seems weaker than could perhaps be expected from the findings that fish is an important source of long-chain n-3 fatty acids that reduce the risk for depression [11,12].

On the whole, our analysis of food items indicates that avoidance of meat consumption is (positively) associated with mental disorders. Since established biological mechanisms do not explain this pronounced association, one could speculate that the phenomenon may be attributable to psychological mechanisms. This interpretation is further supported by our results on the temporal sequencing of the start of vegetarian diet and age of onset of mental disorders. For depressive disorders, anxiety disorders, and somatoform disorders and syndromes we found that on average the adoption of the vegetarian diet follows the onset of mental disorders. Although differences in nutrition status before the actual start of the vegetarian diet affecting vulnerability to mental disorders cannot be ruled out completely, our temporal finding is more consistent with the view that psychological mechanisms cause the associations between vegetarian diet and mental disorders.

Two possible causal mechanisms seem possible. First, because the start of a vegetarian diet, on average, follows the onset of disorder, the experience of a mental disorder may increase the probability of choosing a vegetarian diet (i.e., the mental disorder *causes* the vegetarian diet). Individuals with a history of a mental disorder may exhibit more perceived health-oriented behavior in order to positively influence the course of their disease. Moreover, the experience of a mental disorder may sensitize individuals to the suffering of other living beings, including animals. In addition, elevated levels of health-related anxiety may lead individuals with mental disorders to choose a vegetarian diet as a form of safety or self-protective behavior, because a meat free diet is perceived as more healthy.

Second, a relatively stable psychological mechanism (a third variable) may increase the probability of mental disorders and independently increase the likelihood of choosing a vegetarian diet. The possibility is appealing that psychological mechanisms like the tendency to experience and regulate negative emotions [45,46], high levels of responsibility and perfectionism [47], or contrasting social values of vegetarians [48] might be responsible the pattern of results. However, such possible psychological mechanisms cannot easily explain the temporal sequencing of disorders developing before vegetarian diet.

Health, ethical, environmental, and spiritual reasons are the most important motives for choosing a vegetarian diet [25]. It is possible that subtypes of vegetarians (e.g., health motivated vegetarians vs. ethically motivated vegetarians) may psychologically differ and show different associations with mental disorders. Correspondingly, future research should be mindful of the fact that vegetarians have various motivations for choosing their diet and should clarify whether certain subtypes may be more obviously associated with mental disorders while other subtypes could even be associated with positive mental health.

Although only a small number of individuals in our sample fulfilled lifetime criteria of an eating disorder (anorexia or bulimia, n = 36), we found a strong association with a vegetarian diet. Interestingly, when examining the pattern regarding the age of onset, vegetarian diet seems to have a different significance in eating disorders compared to other mental disorders; the onset of an eating disorder and adoption of a vegetarian diet are much closer in time. This is consistent with evidence that a change in diet often precedes the onset of an eating disorder [29,49]. According to our data the decision to choose a vegetarian (low fat) diet is as likely to precede as to follow the onset of eating disorders.

The relatively simple one-item measure of vegetarian diet presents a limitation to our study. Because vegetarians exhibit a wide diversity of dietary practices, future research should more carefully define vegetarian diet to enable closer examination of the associations between diet and risk of mental disorders and to be able to compare uniform definitions in multiple studies. On the background of the relatively high percentage of vegetarians consuming fish in our study, it seems especially advisable to consider fish consumption in the definition of vegetarian diet more explicitly. Moreover, analysis could benefit from the inclusion of additional measures of nutrition status (e.g. long-chain n-3 fatty acids, vitamin B_12_ levels).

Furthermore, research on cross-cultural differences in vegetarian diet and prevalence rates of mental disorders would be useful. The social situation of vegetarians in Western societies, where vegetarians belong to a minority whose values diverge from the majority, may well differ from those in cultures or subcultures where vegetarianism is a widespread and/or highly valued practice. Results of the study by Beezhold and colleagues [30] with Seventh Day Adventists indicate that a vegetarian diet in a subculture valuing this practice may even be associated with improved levels of mental health.

## Conclusions

On the whole, our results strongly corroborate the past findings in smaller samples of adolescents and young adults, which have demonstrated that in contrast to physical health, a vegetarian diet is not associated with better mental health. Whether compared with a control group of non-vegetarians matched for important socio-demographic characteristics, or with non-vegetarians in general, vegetarians show elevated prevalence rates of diverse mental disorders. Importantly, we found no evidence for a causal role of vegetarian diet in the etiology of mental disorders. Rather, our results are more consistent with the view that the experience of a mental disorder increases the probability of choosing a vegetarian diet, or that psychological factors influence both the probability of choosing a vegetarian diet and the probability of developing a mental disorder.

## Abbreviations

CI: Confidence interval; DSM-IV: Diagnostic and Statistical Manual of Mental Disorders (4th ed); GHS-MHS: German Health Interview and Examination Survey and its Mental Health Supplement; M-CIDI: Composite International Diagnostic Interview; OR: odds ratio.

## Competing interests

The authors declare that they have no competing interests.

## Authors' contributions

JM conceptualized the study, analyzed the data and drafted most parts of the manuscript. XCZ conducted the matching procedure for the non-vegetarian group. FJ participated in the design of the study and revised the manuscript critically for important intellectual content. All authors read and approved the final manuscript.

## Author Information

Johannes Michalak, University of Hildesheim (Germany); Frank Jacobi, Berlin School of Psychology (Germany ) and Technische Universität Dresden (Germany); Xiao Chi Zhang, Ruhr-University Bochum (Germany).
